# Sonic hedgehog signalling as a potential endobronchial biomarker in COPD

**DOI:** 10.1186/s12931-020-01478-x

**Published:** 2020-08-07

**Authors:** Julien Ancel, Randa Belgacemi, Jeanne-Marie Perotin, Zania Diabasana, Sandra Dury, Maxime Dewolf, Arnaud Bonnomet, Nathalie Lalun, Philippe Birembaut, Myriam Polette, Gaëtan Deslée, Valérian Dormoy

**Affiliations:** 1 University of Reims Champagne-Ardenne, Inserm, P3Cell UMR-S 1250, SFR CAP-SANTE, 45 rue Cognacq-Jay, 51092 Reims, France; 2 Department of Pulmonary Medicine, University Hospital of Reims, Hôpital Maison Blanche, 51092 Reims, France; 3 Platform of Cellular and Tissular Imaging (PICT), 51097 Reims, France; 4 University Hospital of Reims, Hôpital Maison Blanche, Laboratoire de Biopathologie, 51092 Reims, France

**Keywords:** Chronic obstructive pulmonary disease, Hedgehog signalling pathway, Airway epithelial cells, Bronchoscopy

## Abstract

**Background:**

The hedgehog (HH) pathway has been associated with chronic obstructive pulmonary disease (COPD) in genome-wide association studies and recent studies suggest that HH signalling could be altered in COPD. We therefore used minimally invasive endobronchial procedures to assess activation of the HH pathway including the main transcription factor, Gli2, and the ligand, Sonic HH (Shh).

**Methods:**

Thirty non-COPD patients and 28 COPD patients were included. Bronchial brushings, bronchoalveolar lavage fluid (BALF) and bronchial biopsies were obtained from fiberoptic bronchoscopy. Characterization of cell populations and subcellular localization were evaluated by immunostaining. ELISA and RNAseq analysis were performed to identify Shh proteins in BAL and transcripts on lung tissues from non-COPD and COPD patients with validation in an external and independent cohort.

**Results:**

Compared to non-COPD patients, COPD patients exhibited a larger proportion of basal cells in bronchial brushings (26 ± 11% vs 13 ± 6%; *p* < 0.0001). Airway basal cells of COPD subjects presented less intense nuclear staining for Gli2 in bronchial brushings and biopsies (*p* < 0.05). Bronchial BALF from COPD patients contained lower Shh concentrations than non-COPD BALF (12.5 vs 40.9 pg/mL; *p* = 0.002); SHH transcripts were also reduced in COPD lungs in the validation cohort (*p* = 0.0001).

**Conclusion:**

This study demonstrates the feasibility of assessing HH pathway activation in respiratory samples collected by bronchoscopy and identifies impaired bronchial epithelial HH signalling in COPD.

## Background

Chronic obstructive pulmonary disease (COPD) is the third leading cause of death worldwide and represents a major public health problem [1]. Tobacco smoke is known to be the main risk factor, leading to epithelial and subepithelial bronchial remodelling [2], including airway basal cell progenitor alterations [3]. These morphological changes result in non-reversible chronic airflow limitation. The pathophysiology of bronchial epithelial remodelling in COPD is complex, heterogeneous and poorly understood, and its impact on clinical phenotypes remains to be elucidated. Due to the incomplete understanding of the mechanisms leading to epithelial remodelling, no treatment targeting epithelial remodelling in COPD is currently available.

Identification of novel pathways involved in COPD pathophysiology constitutes a challenge in order to personalize patient management [4]. A genome-wide association study identified major susceptibility loci in COPD [5], including enrichment of genes related to signalling events mediated by the HH family [6]. Sonic HH signalling is essential for embryonic lung development, regulating epithelial/mesenchymal cell interactions in airways and parenchyma [7, 8]. HH signalling occurs upon Shh ligand binding and leads to Gli2 nuclear translocation, mainly described as an activator of transcription [9]. HH pathway signalling alterations have been observed in respiratory diseases such as pulmonary fibrosis [10, 11], asthma [12, 13] and lung cancer [14]. We recently demonstrated that the HH pathway was inextricably linked to airway epithelial cell (AEC) differentiation in adulthood [15]. Interestingly, in vitro interference with HH induced COPD-like epithelial remodelling, suggesting that Shh signalling may be deficient in COPD. However, assessment of the HH pathway in clinical practice remains challenging.

Using minimally invasive endobronchial procedures, we assessed the HH pathway in COPD and non-COPD patients and demonstrated significant impairment of the HH pathway in COPD.

## Methods

### Study population

**Fig. 1:**
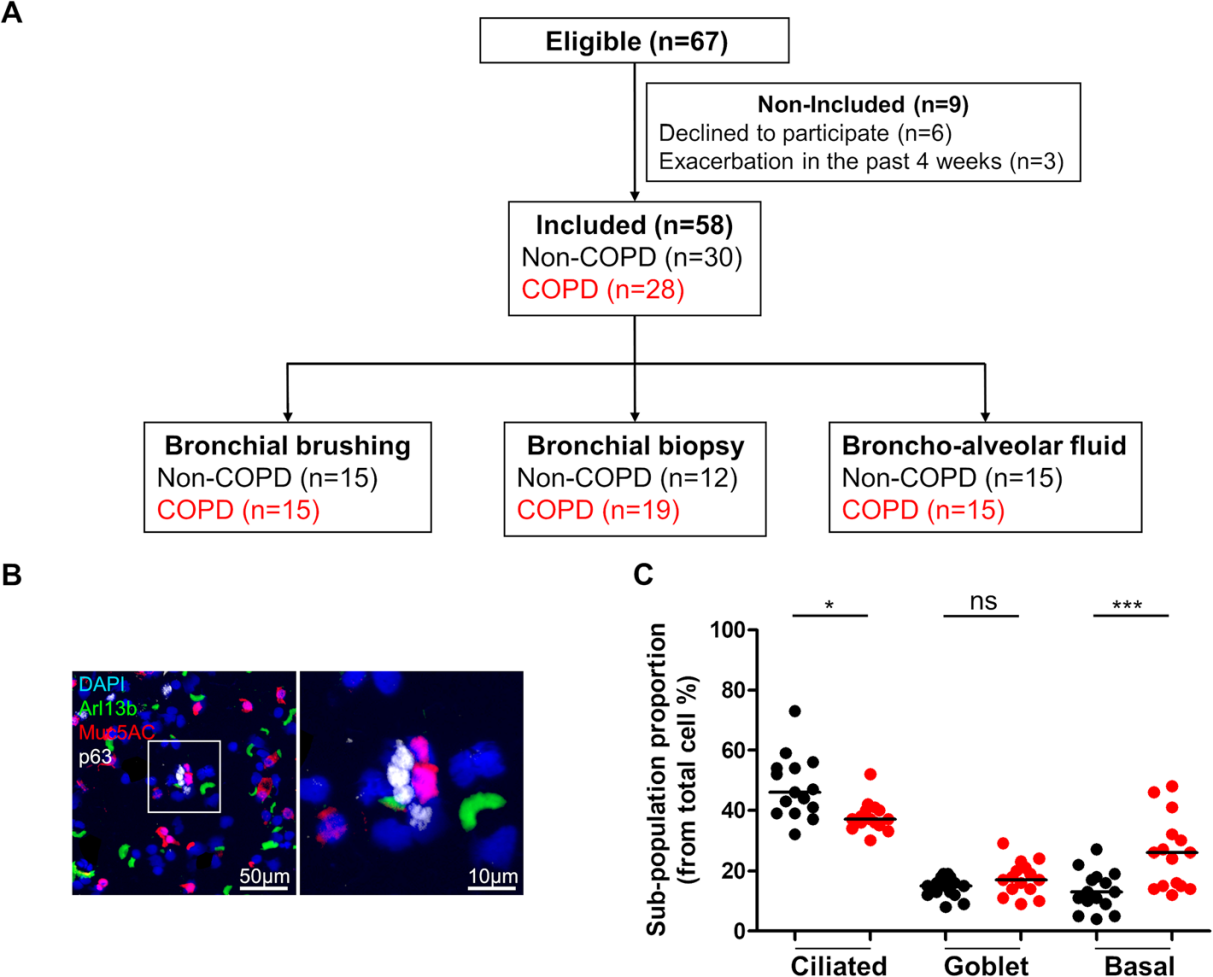
Airway progenitor basal cell population is enriched in COPD. **a**. Study flow chart. **b**. Representative micrograph showing a Region of Interest (ROI) containing AEC obtained by bronchial brushing in a non-COPD patient stained for cilia (Arl13b, green); mucins (Muc5ac, red); basal cells (p63, white) and cell nuclei (DAPI, blue). Magnification corresponding to the selected area is shown. C. Dot plot with median showing the percentage of ciliated, goblet and basal cells in both non-COPD (*n* = 15) (black circle) and COPD patients (*n* = 15) (red circle). *, *p* < 0.05 and ***, *p* < 0.0001; non-COPD vs COPD

COPD and non-COPD control subjects (Fig. 1a) were prospectively recruited from the Department of pulmonary medicine, University Hospital of Reims (France). Non-COPD patients (*n* = 30) with no diagnosis of chronic respiratory disease were recruited from the Department of pulmonary medicine. COPD patients (*n* = 28) were enrolled on the basis of clinical and functional assessments with a forced expiratory volume in 1 s (FEV_1_)/forced vital capacity (FVC) < 0.7 after bronchodilation. At inclusion, all patients were stable with no acute exacerbation of COPD for 4 weeks. Patients with asthma, cystic fibrosis, tuberculosis, cancer, or other chronic respiratory disease were excluded. Patient characteristics, including demographic data, medical history, treatments, respiratory symptoms and pulmonary function tests (PFT), were collected using a predefined case report form. Subjects who had ceased smoking for more than 6 months were considered to be ex-smokers. The severity of COPD was determined according to the spirometric classification (GOLD 1: FEV_1_ ≥ 80% predicted, GOLD 2: 50% ≤ FEV_1_ < 80% predicted, GOLD 3: 30% ≤ FEV_1_ < 50% predicted, GOLD 4: FEV_1_ < 30% predicted) [16]. Frequent exacerbations were defined as at least 2 exacerbations in the past 12 months [16, 17]. All subjects provided their written informed consent to the study (Research and Innovation in Chronic Inflammatory Respiratory Diseases-RINNOPARI, NCT02924818).

All subjects underwent fiberoptic bronchoscopy with bronchial brushings, biopsies and/or bronchoalveolar lavage (BAL) under routine clinical conditions according to international guidelines [18].

### Sample processing

Fresh airway epithelial cells (AEC) obtained from bronchial brushings (right lower lobe) were suspended within 30 min in RPMI (1% penicillin/streptomycin+ 10% BSA) before centrifugation (12,500 rpm twice). The cell pellet was dissociated in 1 mL of Trypsin-Versene® and centrifuged (12,500 rpm twice). Cells were counted using a haemocytometer. Microscopy slides were coated with 100 μL of 3% PBS-BSA by centrifugation (10,000 rpm for 10 min) and 25,000 AEC were then centrifuged (750 rpm for 8 min). Cells were immediately fixed with ice-cold methanol and stored at 4 °C until immunofluorescence analysis.

Bronchial biopsies (3rd to 5th order) were fixed in 4% formalin for 24 h. Three μm sections were cut from formalin-fixed paraffin-embedded (FFPE) blocks and processed for H&E staining and observed under the microscope (× 20) to confirm the presence of epithelium. Suitable FFPE bronchial section slides were deparaffinised before immunofluorescence staining.

BALF was sampled by fractionating aliquots of bronchial (first 50 mL) and alveolar samples (100 mL) centrifuged twice at 1300 rpm for 5 min [19]. Supernatants were collected and stored at − 80 °C until ELISA analysis.

### Immunofluorescence staining

Samples were rehydrated with PBS and blocked with 10% PBS-BSA for 30 min at room temperature before incubation with the following primary antibodies overnight at 4 °C in 3% PBS-BSA: rabbit anti-Arl13b (17711–1-ap, ProteinTech, 1:200); mouse anti-Muc5ac (NBP2–15196, Novus Biologicals, 1:100); mouse anti-Acetylated-α-tubulin (T6793, Sigma Aldrich, 1:1000); goat anti-p63 (AF1916, R&D systems, 1:100); rabbit anti-pancytokeratin (E-AB-33599; Clinisciences, 1:100); mouse anti-vimentin (M0725; Dako, 1:100); rabbit anti-Gli1 (HPA065172, Sigma Aldrich, 1 μg/mL); rabbit anti-Gli2 (HPA074275, Sigma Aldrich, 0,4 μg/mL); goat anti-Shh (AF464, R&D systems, 1:100); rabbit anti-Ptch1 (E-AB-10571, Clinisciences, 1:100); mouse anti-Hhip (WH0064399M1, Sigma-Aldrich, 1:100). Samples were washed with PBS and incubated with the appropriate secondary antibodies in PBS-BSA 3% for 1 h at room temperature. DNA was stained with DAPI (1:1000) during incubation with secondary antibodies. Negative controls were performed by omitting the primary antibody or by incubating with a corresponding IgG isotype.

Cells and bronchial sections were semi-automatically digitized into virtual slides using a VS120 virtual slide microscope at 20x magnification (NA 0.75) (Olympus, Tokyo, Japan) and consulted with OlyVIA™ viewer (Olympus). Cytological integrity and delimitation of individual cells were verified by light microscopy. Three regions of interest (1000*1000 pixels by ROI) were randomly extracted by BIOP VSI reader export plug-in (https://biop.epfl.ch/TOOL_VSI_Reader.html) and processed with ImageJ (National Institutes of Health) for analysis (450 cells were counted per slide).

### Shh enzyme-linked ImmunoSorbent assay

Shh protein concentrations in BALF were assayed by enzyme-linked immunosorbent assay (ELISA) according to RayBiotech instructions (ELH-ShhN-001). The limit of detection of the assay was 8 pg/mL.

### Validation cohort

To validate our observations, we analysed human lung gene expression on an independent cohort (GSE47460) dataset including 145 COPD subjects and 91 control subjects [20, 21].

### Statistical analysis

Data are expressed as mean ± standard deviation (SEM). All continuous variables are represented with dot-plot and median. Associations between features were studied using Chi-square or Fisher’s exact test, as appropriate. A non-parametric Mann-Whitney test was used to analyse differences between experimental conditions and linear regression was performed with the Spearman process. Multiple groups were analysed using the Kruskal-Wallis procedure followed by the Conover and Iman test. To evaluate differences in expression levels between candidate genes, we used the Mann-Whitney *U* test for independent samples of the normalized log2-transformed microarray expression values. In all exploratory analyses, results with two-sided *p*-value ≤0.05 were considered significant. XLSTAT software (Addinsoft Company, Paris, France) was used to analyse and format data.

## Results

### Patient characteristics

We included 30 non-COPD patients and 28 COPD patients to investigate HH pathway alterations in bronchial brushings, biopsies and BALF (Fig. 1a). The two groups did not differ in terms of age or sex (Table 1). As expected, COPD patients were more frequently current smokers and were characterized by higher smoking exposure, dyspnoea and airflow limitation. Twenty (71%) COPD patients had at least one inhaled treatment. Nineteen (68%) patients frequently used LABA, while 14 (50%) and 13 (46%) were treated with LAMA and ICS, respectively.

**Table 1:** Baseline characteristics of the population

	Non-COPD (*n* = 30)	COPD (*n* = 28)	*p*-value
Sex ratio H/F	13/17	17/11	ns
Age (years)	53.9 ± 15.3	62.1 ± 10.6	ns
Smoking history			0.009
Never smokers	8 (27%)	0	
Current-smokers	12 (40%)	12 (43%)	
Former-smokers	10 (33%)	16 (57%)	
Pack-years	21 ± 21	41 ± 22	< 0.001
Spirometry			
FEV_1_, % of predicted value	97 ± 19	55 ± 25	< 0.0001
FVC, % of predicted value	100 ± 18	82 ± 20	0.003
FEV_1_/FVC %	81 ± 9	49 ± 12	< 0.0001
Spirometric GOLD 1/2/3/4	NA	7/6/9/6	–
GOLD ABCD (mMRC)	NA	8/6/6/8	–
GOLD ABCD (CAT)	NA	7/7/4/10	–
Frequent exacerbation (> 1/year)	–	10 (36%)	–

Data are expressed as mean ± SD or number (%) *FEV*_*1*_ forced expiratory volume in 1 s, *FVC* forced vital capacity, *mMRC* modified medical research council, *CAT* COPD assessment test

ns: non-significate

### Percentages of ciliated and basal AEC populations are altered in COPD patients

Epithelial cell populations collected by bronchial brushing were characterized by immunostaining for ciliated (Arl13b+), goblet (Muc5ac+) and basal (p63+) cell markers (Supplemental Table 1 and Fig. 1b). Compared to non-COPD subjects, COPD subjects had lower percentages of ciliated cells (37 ± 5% vs 48 ± 10%; *p* = 0.045) and higher percentages of basal cells (26 ± 11% vs 13 ± 6%; *p* < 0.0001), while similar percentages of goblet cells were observed in the non-COPD and COPD groups (15 ± 3% vs 17 ± 5%; *p* = 0.154) (Fig. 1c).

### Gli2 is reduced in nuclear basal cells progenitors from COPD patients

**Fig. 2:**
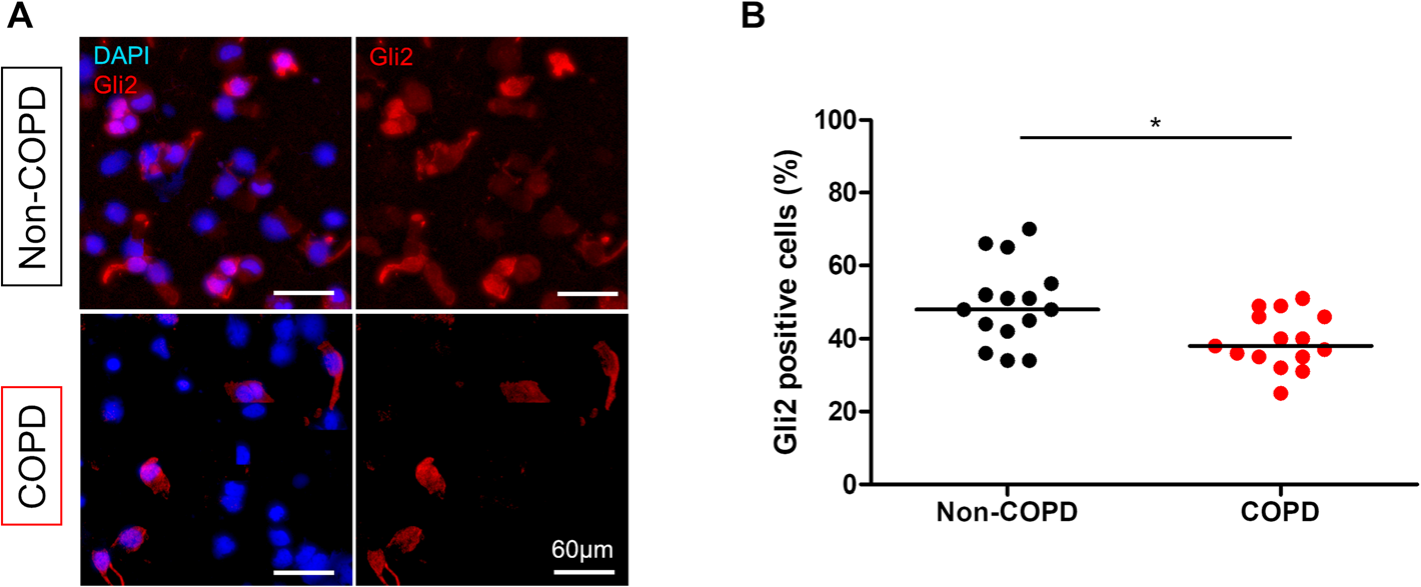
Gli2 expression is decreased in AEC from COPD patients. **a**. Representative micrograph showing a ROI of a bronchial brushing stained for Gli2 (Gli2, red) and cell nuclei (DAPI, blue) in both non-COPD (upper panel) and COPD patients (lower panel). Magnification corresponding to the selected area is shown. **b**. Dot plot with median showing the total percentage of Gli2-positive cells in non-COPD (n = 15) and COPD patients (*n* = 15). *, *p* < 0.05

**Fig. 3:**
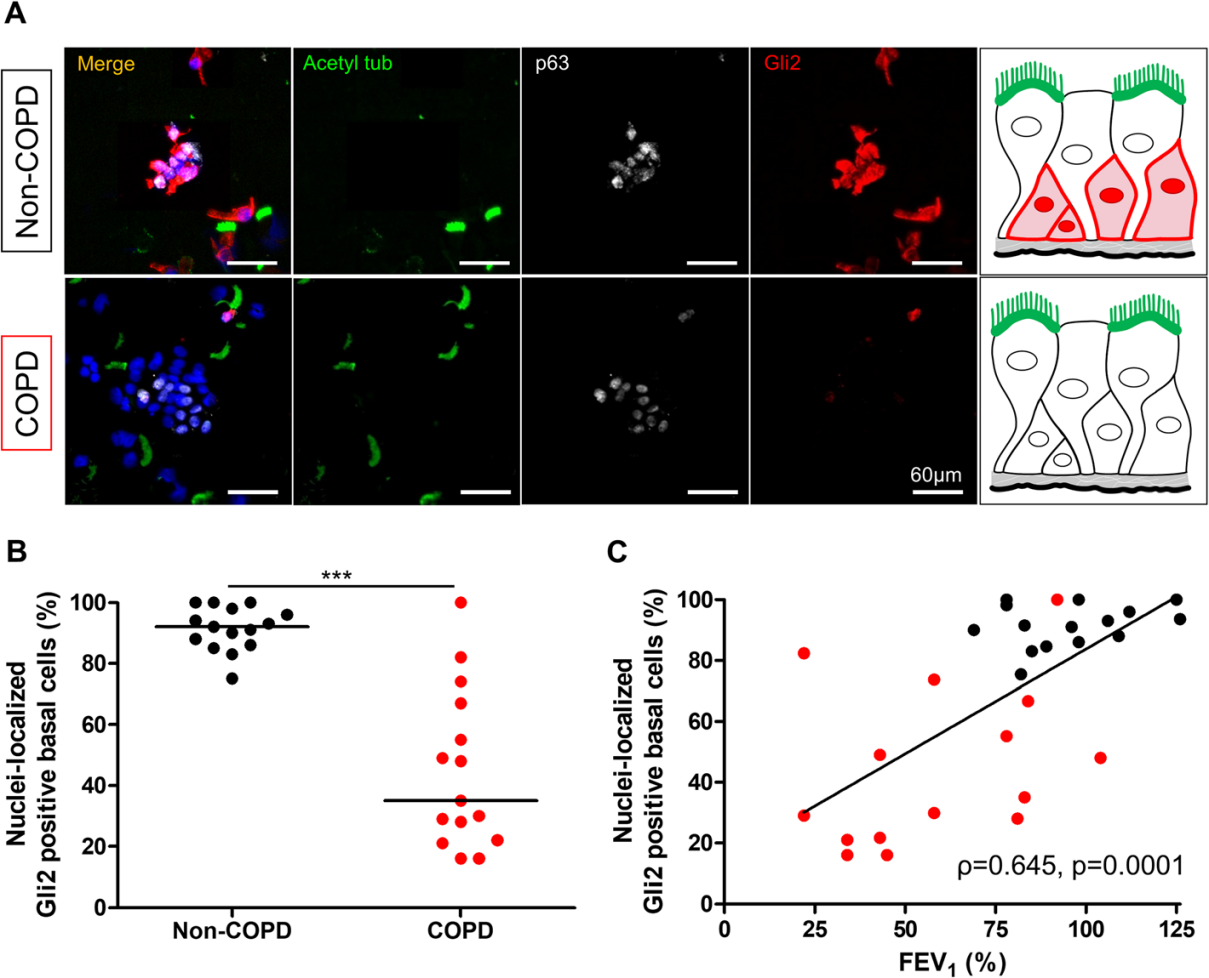
Gli2 expression is decreased in airway progenitor basal cell nuclei from COPD patients. **a**. Representative micrograph showing a ROI of a bronchial brushing stained for cilia (Acetylated tubulin, green); Gli2 (Gli2, red); basal cells (p63, white) and cell nuclei (DAPI, blue) in both non-COPD (upper panel) and COPD patients (lower panel). Magnification corresponding to the selected area is shown. Insets depict localization of the Gli2 transcription factor. **b**. Dot plot with median showing the percentage of Gli2-positive basal cell nuclei in non-COPD (*n* = 15) and COPD patients (n = 15). ***, *p* < 0.0001. **c**. Linear regression of the percentages of Gli2-positive basal cell nuclei according to FEV_1_ (% predicted) for non-COPD (n = 15) and COPD patients (n = 15). Non-COPD patients are represented by black circles and COPD patients are represented by red circles

The number of Gli2-positive cells nuclei in total AEC obtained by bronchial brushing was decreased in the COPD group compared to the non-COPD group: 39% vs 49% of total AEC; *p* = 0.017 (Fig. 2a and b). Focusing solely on basal cells, the number of Gli2-positive cell nuclei in basal cells was also decreased in the COPD group compared to the non-COPD group: 44% vs 91% of basal cells (mean, *p* < 0.0001; Fig. 3a and b). We identified two different patterns of Gli2 cellular localization in COPD subjects: either complete loss of the transcription factor or cytoplasmic-restricted localization (Supplemental Figure 1).

Lower Gli2 nuclear staining in basal cells was associated with lower FEV_1_ (*ρ* = 0.645, *p* = 0.0001, Fig. 3c) and lower FEV_1_/FVC ratio (*ρ* = 0.737, *p* < 0.0001, Supplemental Figure 2A). No association was found between Gli2 nuclear staining and inhaled treatments, smoking history or clinical features (Supplemental Figure 2B).

### Alteration of Gli2 expression in bronchial epithelium and stroma from COPD patients

**Fig. 4:**
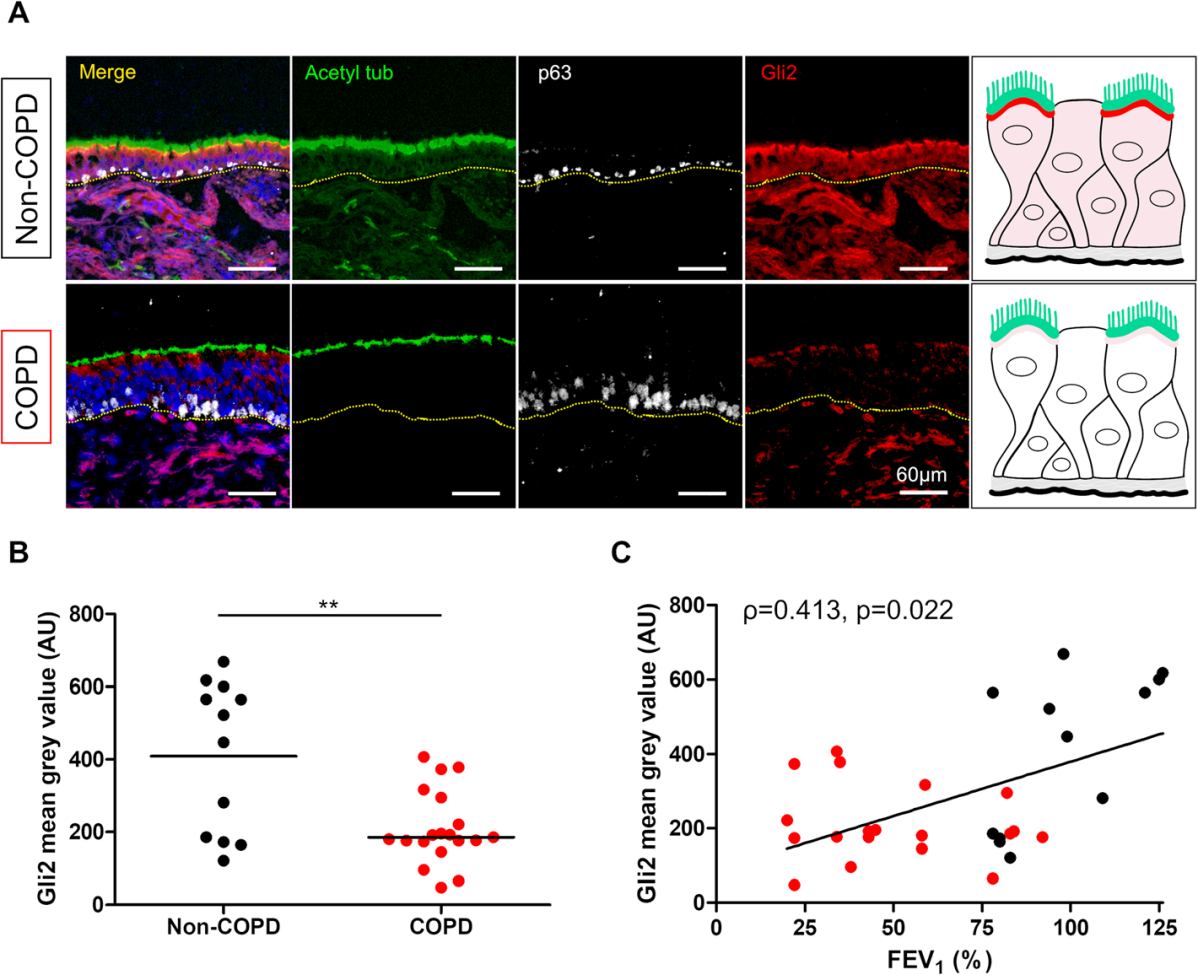
Gli2 transcription factor is decreased in whole bronchial epithelium from COPD patients. **a**. Representative micrograph showing a ROI of a bronchial biopsy stained for cilia (Acetylated tubulin, green); Gli2 (Gli2, red); basal cells (p63, white) and cell nuclei (DAPI, blue) in both non-COPD (upper panel) and COPD patients (lower panel). Magnification corresponding to the selected area is shown. Insets depict localization of the Gli2 transcription factor. **b**. Dot plot with median showing the intensity of Gli2 mean grey value (Arbitrary units, AU) in whole bronchial epithelium in non-COPD (*n* = 12) and COPD patients (*n* = 19). **, *p* < 0.001 C. Linear regression of the intensity of Gli2 mean grey value according to FEV_1_ (% predicted) in non-COPD (n = 12) and COPD patients (n = 19). Non-COPD patients are represented by black circles and COPD patients are represented by red circles

We completed our approach by comparing HH components in bronchial biopsies. The material obtained by bronchial biopsies was situated more proximally than obtained by bronchial brushing, providing access to intact epithelia (Supplemental Table 2). The Gli2 distribution at this superior hierarchical airway branching was more diffuse, but a two-fold decrease of AEC Gli2 staining in bronchial epithelium was observed in the COPD group compared to the non-COPD group (*p* = 0.008, Fig. 4a and b). As observed in AEC obtained by bronchial brushing, decreased Gli2 staining in bronchial epithelium was associated with lower FEV_1_ (*ρ* = 0.413, *p* = 0.022; Fig. 4c) and lower FEV_1_/FVC ratio (*ρ* = 0.411, *p* = 0.022; Supplemental Figure 3).

Since HH pathway homeostasis may rely on molecular crosstalks between stromal populations and AEC [10, 22–24], we assessed HH mesenchymal response in peribronchial tissues (Supplemental Figure 4). Mesenchymal cells (stained for vimentin) were sparsely distributed in the stroma, and we observed differences in terms of activation between tissues from non-COPD and COPD patients. Few mesenchymal cells were Gli1+ in both groups, and we observed a gradient of Gli2+ cells in the stroma from the epithelial layer to the adventitia. Gli2+ mesenchymal cells were also decreased in the stroma of COPD patients.

### Reception of Shh signalling is altered in COPD patients

**Fig. 5:**
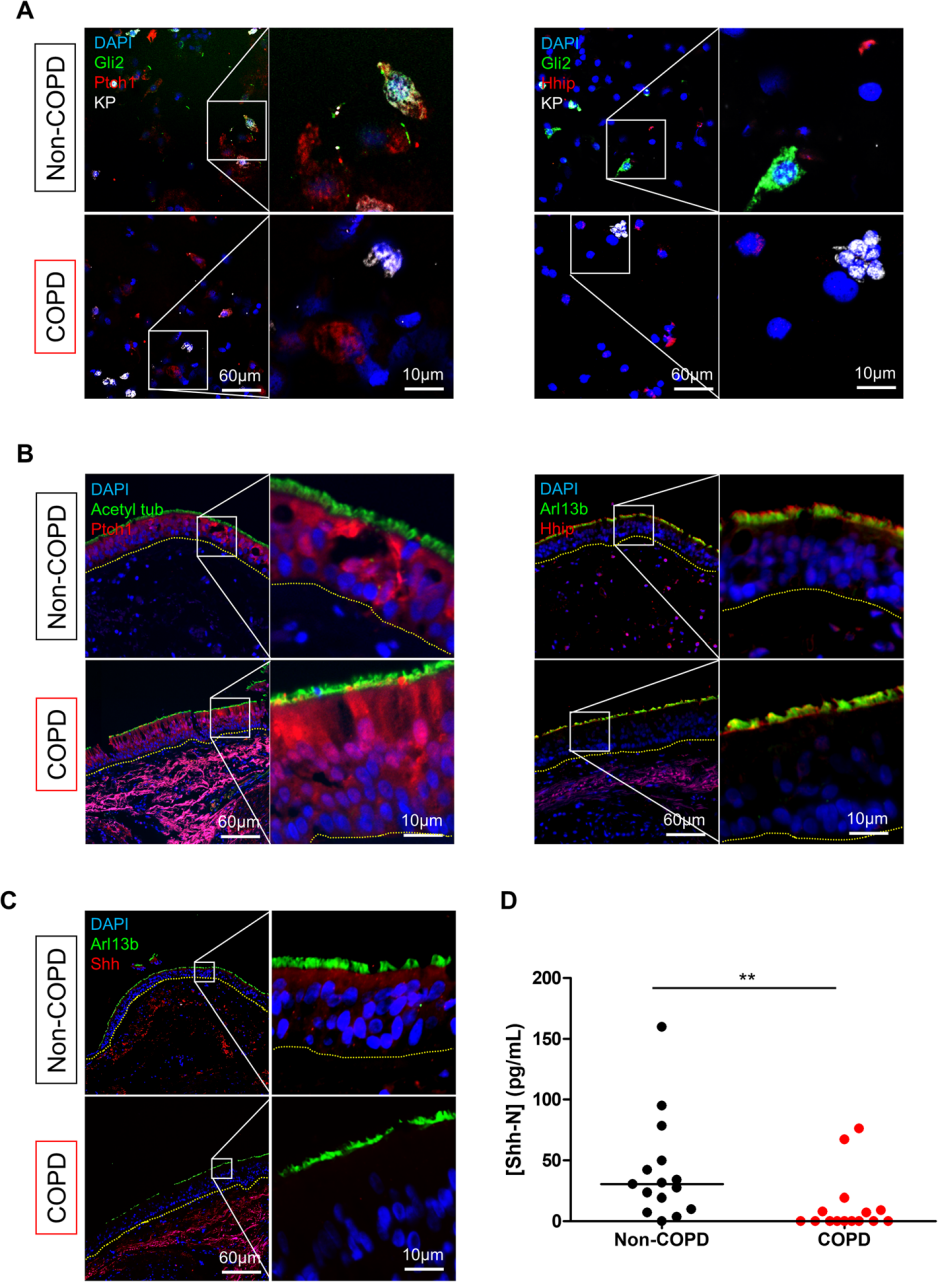
Shh activating ligand is deficient in COPD bronchi. **a**. Representative micrograph showing a ROI of a bronchial brushing stained for basal cells (pancytokeratin, KP, white); Gli2 (green); Ptch1 (red, left panel); Hhip (red, right panel); and cell nuclei (DAPI, blue). Magnification corresponding to the selected area is shown. **b**. Representative micrograph showing a ROI of a bronchial biopsy stained for cilia (Acetylated tubulin or Arl13b, green); Ptch1 (red, left panel); Hhip (red, right panel); and cell nuclei (DAPI, blue). Magnification corresponding to the selected area is shown. **c**. Representative micrograph showing a ROI of a bronchial biopsy stained for cilia (Arl13b, green); Shh (red) and cell nuclei (DAPI, blue). Magnification corresponding to the selected area is shown. **d**. Dot plot with median representing Shh concentrations measured by ELISA in bronchial BALF from non-COPD (n = 15) and COPD patients (n = 15). **, *p* < 0.001

To identify a potential mechanism involved in the Shh pathway dysregulation observed in COPD, we first investigated the localizations of the two main receptors of the ligand: Ptch1 and Hhip. In AEC (Fig. 5a), Ptch1 stained non-differentiated and differentiated cells, in which it was also found to be associated with Gli2 staining, suggesting that both cell populations may transduce Shh signalling. On the contrary, Hhip was not found on non-differentiated cells. No difference in terms of these two Shh receptors was observed between non-COPD and COPD patients. Immunostaining of bronchial epithelium (Fig. 5b) confirmed the findings observed on isolated AEC, with epithelial cytoplasmic localization for Ptch1 and cilia-associated localization for Hhip. Interestingly, no difference in terms of epithelial localizations of Ptch1 and Hhip was observed between non-COPD and COPD patients, whereas mesenchymal populations appeared to express Hhip in non-COPD tissues.

Due to the loss of cytoarchitecture in bronchial brushing samples, we also characterized Shh localization in bronchial biopsies (Fig. 5c). Shh appeared to be predominantly present at the apical surface of ciliated cells in non-COPD tissues, but was absent in COPD epithelia. We then separately analysed bronchial fluid (corresponding to the fluid collected from the first 50 mL of saline injected during BAL) and alveolar fluid (corresponding to the fluid collected from the last 100 mL of saline injected during BAL) (Fig. 5d and Supplemental Figure 5A) from 15 COPD and 15 non-COPD subjects (Supplemental Table 3) and quantified the SHH pathway-activating ligand Shh by ELISA. We observed a dramatic reduction of Shh protein concentration in bronchial samples from COPD patients compared to non-COPD subjects (12.5 vs 40.9 pg/mL; *p* = 0.002; Fig. 5d). Shh protein was not detectable in 67% (*n* = 10) of samples from COPD patients and in 20% (*n* = 3) of samples from non-COPD subjects.

Analysis of alveolar BALF samples did not reveal any differences in Shh protein concentrations between COPD and non-COPD groups (17.6 vs 28.5 pg/mL, respectively, *p* = 0.228; Supplemental Figure 5A). In addition, there was no significant difference in terms of Shh concentrations among COPD patients according to inhaled corticosteroid treatment (Supplemental Figure 5B).

### Shh ligand is deficient in COPD lung tissues

**Fig. 6:**
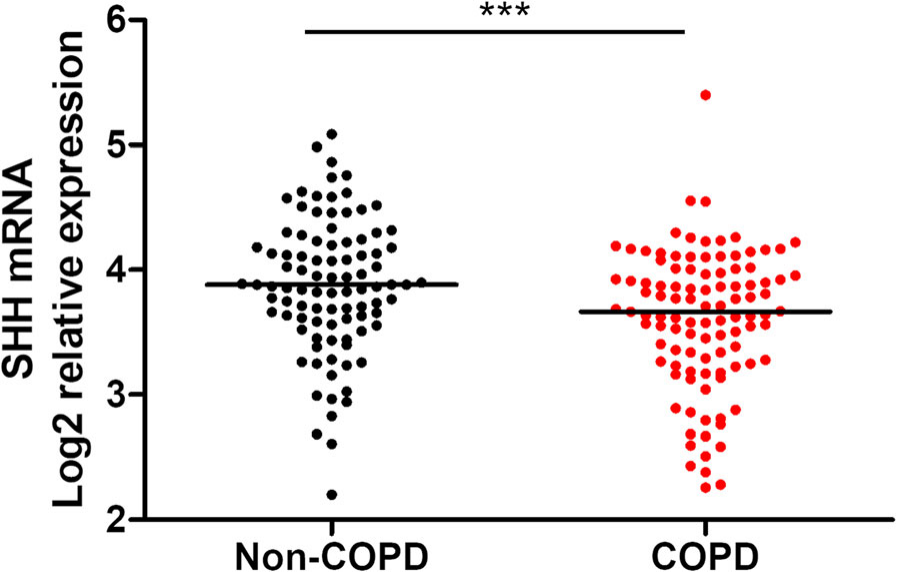
Shh transcript levels are decreased in COPD lung tissues. Dot plot with median showing normalized expression of Log2-transformed SHH expression in lung tissue from non-COPD (*n* = 91) and COPD patients (*n* = 145). ***, *p* < 0.0001. Transcript expression microarrays were extracted from the GSE47460 dataset available at (https://www.ncbi.nlm.nih.gov/gds). Non-COPD patients are represented by black circles and COPD patients are represented by red circles

To validate our results in an independent cohort, we then analysed a transcriptomic dataset obtained by RNA sequencing of lung tissue in COPD and non-COPD subjects (GSE47460). We confirmed a significant reduction in SHH gene expression in the COPD group compared to the non-COPD group (3.666 vs 3.883, log2 relative expression, *p* = 0.0001; Fig. 6). SHH gene expression was not associated with smoking history, inhaled treatment or clinical features.

## Discussion

In this prospective translational study, we demonstrated a marked dysregulation of the HH pathway in COPD patients by means of minimally invasive endobronchial procedures allowing the collection and analysis of several complementary biological samples. This approach provided access to cancer-free patients, thereby avoiding a major confounding bias in HH evaluation and allowed us to categorize patients with mild to very severe COPD, providing a representative range of severity.

Using this approach, we confirmed alterations of AEC proportions in COPD, including an increase in basal cells and a dramatic reduction of the number of ciliated cells, as previously described [3], suggesting an impaired airway basal cell progenitor differentiation process [25]. Ideally, this basic quantification could be completed by cellular characterization using additional cell markers, possibly resulting in a single cell sequencing approach, such as the recent findings obtained in the fields of in vitro AEC differentiation, idiopathic pulmonary fibrosis or asthma [26–30]. Our recently published results suggested a role of the HH pathway in dysregulation of basal cell differentiation that could result in epithelial remodelling, a key feature of COPD [15].

To further explore this hypothesis, we then focused on the critical molecular players responsible for the biological effects of the HH pathway: the activating ligand, Shh, its main receptor Ptch1, its main co-receptor Hhip, and its main transcription factor Gli2, while previous studies have exclusively focused on analysis of the Ptch1 ligand receptor [31]. We first identified a marked decrease in Gli2 nuclear localization of basal cells and Gli2 protein levels in bronchial epithelium, highlighting a defect in HH signalling in COPD. The observed decrease of Gli2+ nuclear staining of cells obtained by bronchial brushing was consistent with our previous findings [15]. The apparent discrepancy with the staining observed on bronchial biopsies could be explained by the different sources of biological material. Moreover, accumulation of Gli2 on the apical cell surface may facilitate rapid activation of the HH pathway in these cells.

HH signalling partially orchestrates mesenchymal cell homeostasis during organogenesis and cellular crosstalks may be altered, as in idiopathic pulmonary fibrosis [10]. Our preliminary observations in stroma need to be confirmed by further investigations, as active proliferation in response to Shh signalling may occur exclusively during the process of connective tissue remodelling.

In concordance with our recent observations on FFPE tissues from lung resections [15], Ptch1, the receptor responsible for transduction of Shh signalling, was also found on non-differentiated and differentiated cells from isolated AEC and biopsies. Gli2 may be found in basal AEC nuclei, but can also be observed subciliary in ciliated cells, suggesting the presence of autocrine and paracrine signalling in the epithelium. We then focused on Hhip, another Shh receptor acting as an inhibitor of HH signalling, which is particularly interesting, as genetic alteration of Hhip was associated with COPD and emphysema in genome-wide associated studies and murine models [4, 5, 31–35]. We detected Hhip in differentiated cells, but the absence of differential Hhip localization between non-COPD and COPD samples suggested that genetic alterations of Hhip may impact protein function rather than protein expression.

We also analysed the SHH pathway-activating ligand Shh in bronchial biopsies and observed a loss of Shh on the apical surface of ciliated cells, which was confirmed in bronchial fluids, in which Shh ligand concentrations were decreased in COPD subjects. Altogether, our results suggest dysregulation of the SHH pathway either at the level of the production of the ligand or affecting the activating/inhibiting receptor binding balance in COPD.

This result was confirmed using the lung transcriptome dataset from of an independent COPD cohort. Although significant, the minor differences in gene expression levels observed may be due to the sample collection technique, involving extraction of mRNA from both epithelium and subepithelial compartments with enriched HH players, such as fibroblasts [36].

Our results are consistent with our previous observations showing that prevention of ligand-induced HH activation resulted in epithelial remodelling with an increased number of basal cells and decreased ciliogenesis [15], therefore mimicking the remodelling features of COPD observed in endobronchial samples in our study.

HH signalling has been associated with several respiratory diseases, including exacerbation of pulmonary fibrosis [10, 37], asthma [13, 38] or pulmonary arterial hypertension [39]. SHH signalling activation has also been associated with cigarette smoke [40, 41]. In our study, Shh pathway markers were not associated with smoking history or inhaled corticosteroid treatment. A previous high content screening study reported corticosteroid-mediated Shh modification [42]. Budesonide inhibited HH signalling, while other compounds failed to trigger pathway activation, increasing cellular sensitivity to HH ligands and impairing pathway inhibition by co-administered pharmacological antagonists of Smo signalling. The heterogeneity of inhaled corticosteroids and variable glucocorticoid concentrations in airways [43] could explain the absence of any corticosteroid-associated impact on HH activity in our study.

In this study, we confirmed that the HH pathway is clearly deficient in bronchial epithelium from COPD patients. Importantly, HH dysregulations were detected on a wide range of respiratory tract specimens: isolated AEC from bronchial brushing, bronchial biopsies, BAL and lung tissues. We also demonstrated, for the first time, that HH pathway inactivation is associated with the absence of its canonical activating ligand, Shh. Investigating the molecular mechanisms involved in Shh-associated epithelial remodelling may pave the way to characterization of novel markers in order to improve the management of COPD patients.

## Conclusions

Our study, based on endobronchial samplings, confirmed dysregulation of Sonic HH signalling in COPD via the Gli2 transcription factor. More importantly, we demonstrated that HH impairment was related to Shh ligand deficiency in bronchial samples. These results highlight the major implication of HH in COPD. However, the relevance of these findings in terms of phenotyping and a potential innovative therapeutic target need to be further evaluated.

## Supplementary information

**Additional file 1: Table S1.** Baseline characteristics of the population who underwent bronchial brushing.

**Additional file 2: Table S2.** Baseline characteristics of the population who underwent bronchial biopsies.

**Additional file 3: Table S3.** Baseline characteristics of the population who underwent broncho-alveolar lavages.

**Additional file 4: Figure S1.** Heterogeneous Gli2 localization pattern in airway progenitor basal cells from COPD patients. Representative micrograph showing a Region Of Interest of a bronchial brushing stained for cilia (Acetylated tubulin, green); Gli2 (Gli2, red); basal cell (p63, white) and cell nuclei (DAPI, blue) both in COPD. Magnification corresponding to the selected area is shown. Insets depict localization of the Gli2 transcription factor.

**Additional file 5: Figure S2.** Nuclear localized Gli2 is associated with FEV_1_/FVC but not with ICS. A. Linear regression of the percentages of nuclear Gli2-positive basal cells according to FEV_1_/FVC (% predicted) for non-COPD (*n* = 15) and COPD patients (n = 15). B. Dot plot with median, showing no association of the percentage of nuclear localized Gli2-positive basal cells in COPD patients according to inhaled corticosteroid treatment (ICS). Non-COPD patients are represented by black circles and COPD patients are represented by red circles.

**Additional file 6: Figure S3.** Gli2 transcription factor in whole bronchial epithelium is associated to FEV_1_/FVC ratio. A. Linear regression of the intensity of Gli2 mean grey value (Arbitrary Units, AU) in whole bronchial epithelium according to FEV_1_/FVC ratio (% predicted) for non-COPD (*n* = 12) and COPD patients (*n* = 19). Non-COPD patients are represented by black circles and COPD patients are represented by red circles.

**Additional file 7: Figure S4.** Peribronchial mesenchymal cells present no alteration of HH signalling in COPD patients. Representative micrograph showing a ROI of a bronchial biopsy stained for mesenchymal cells (vimentin, green); Gli1 (left, red) or Gli2 (right, red); and cell nuclei (DAPI, blue). Magnification corresponding to the selected area is shown.

**Additional file 8: Figure S5.** Shh activating ligand concentrations in alveolar BALF is not altered in COPD. A. Dot plot with median representing the Shh concentrations measured by ELISA in alveolar BALF from non-COPD (*n* = 15) and COPD patients (n = 15). B. Dot plot with median showing Shh concentrations in COPD patients according to inhaled corticosteroid treatment (ICS).

## Data Availability

All data generated or analysed during this study are available from the corresponding author on reasonable request.

## Abbreviations

COPD: Chronic obstructive pulmonary disease
HH: Hedgehog
SHH: Sonic hedgehog
AEC: Airway epithelial cell
RINNOPARI: Research and innovation in chronic inflammatory respiratory diseases
ROI: Region of interest
PFT: Pulmonary function tests
FEV_1_: Forced expiratory volume in 1-s
FVC: Forced vital capacity
GOLD: Global initiative for chronic obstructive lung disease
BSA: Bovine serum albumin
FFPE: Formalin-fixed paraffin-embedded
ELISA: Enzyme-linked immunosorbent assay
CAT: COPD assessment test
mMRC: modified Medical Research Council
LABA: Long-acting β2-agonist
LAMA: Long-acting muscarinic antagonist
ICS: Inhaled corticosteroid
Gli2: Glioma-associated oncogene 2
Ptch1: Patched 1
