# Supplementary material for: Sonic hedgehog signalling as a potential endobronchial biomarker in COPD
Source: Respir Res. 2020 Aug 7;21:207. doi: 10.1186/s12931-020-01478-x (PMC7412648; doi:10.1186/s12931-020-01478-x)
Supplement: Supplementary file 2 — Additional file 2: Table S2. Baseline characteristics of the population who underwent bronchial biopsies. [file 12931_2020_1478_MOESM2_ESM.docx]

|  | | **Non-COPD**  **(n=12)** | **COPD**  **(n=19)** | **p-value** |
| --- | --- | --- | --- | --- |
| Sex ratio H/F | | 4/8 | 11/8 | ns |
| Age (years) | | 54.2±18.9 | 63.5±8.4 | ns |
| Smoking history | |  |  | ns |
|  | Never smokers | 2 (17%) | 0 |  |
|  | Current-smokers | 5 (42%) | 7 (37%) |  |
|  | Former-smokers | 5 (42%) | 12 (63%) |  |
|  | Pack-years | 19±16 | 46±21 | 0.001 |
| Spirometry | |  |  |  |
|  | FEV_1_, % of predicted value | 98±18 | 50±24 | <0.0001 |
|  | FVC, % of predicted value | 106±19 | 78±20 | 0.002 |
|  | FEV_1_/FVC % | 80±12 | 48±13 | <0.0001 |
| Spirometric GOLD 1/2/3/4 | | NA | 4/4/7/4 | - |
| GOLD ABCD (mMRC) | | NA | 5/3/5/6 | - |
| GOLD ABCD (CAT) | | NA | 3/4/3/9 | - |
| Frequent exacerbation (>1/year) | | - | 7 (37%) | - |

**Supplemental table 2**: Baseline characteristics of the population who underwent bronchial biopsies

Data are expressed as mean ± SD or number (%) FEV_1_: Forced Expiratory Volume in one second; FVC: Forced Vital Capacity; mMRC: Modified Medical Research Council; CAT: COPD Assessment Test.

ns: non-significate.
