# Supplementary material for: Sonic hedgehog signalling as a potential endobronchial biomarker in COPD
Source: Respir Res. 2020 Aug 7;21:207. doi: 10.1186/s12931-020-01478-x (PMC7412648; doi:10.1186/s12931-020-01478-x)
Supplement: Supplementary file 3 — Additional file 3: Table S3. Baseline characteristics of the population who underwent broncho-alveolar lavages. [file 12931_2020_1478_MOESM3_ESM.docx]

|  | | **Non-COPD**  **(n=15)** | **COPD**  **(n=15)** | **p-value** |
| --- | --- | --- | --- | --- |
| Sex ratio H/F | | 7/8 | 11/4 | ns |
| Age (years) | | 53.0±15.9 | 63.1±12.9 | ns |
| Smoking history | |  |  | ns |
|  | Never smokers | 5 (33%) | 0 |  |
|  | Current-smokers | 4 (27%) | 8 (53%) |  |
|  | Former-smokers | 6 (40%) | 7 (47%) |  |
|  | Pack-years | 15±16 | 43±23 | 0.001 |
| Spirometry | |  |  |  |
|  | FEV_1_, % of predicted value | 100±23 | 68±24 | 0.001 |
|  | FVC, % of predicted value | 103±22 | 91±21 | ns |
|  | FEV_1_/FVC % | 82±7 | 55±10 | <0.0001 |
| Spirometric GOLD 1/2/3/4 | | NA | 6/5/3/1 | - |
| GOLD ABCD (mMRC) | | NA | 6/4/3/2 | - |
| GOLD ABCD CAT | | NA | 3/6/1/5 | - |
| Frequent exacerbation (>1/year) | | - | 5 (33%) | - |

**Supplemental table 1**: Baseline characteristics of the population who underwent broncho-alveolar lavages.

Data are expressed as mean ± SD or number (%) FEV_1_: Forced Expiratory Volume in one second; FVC: Forced Vital Capacity; mMRC: Modified Medical Research Council; CAT: COPD Assessment Test.

ns: non-significate.
