# Supplementary figures and images for: Sonic hedgehog signalling as a potential endobronchial biomarker in COPD
Source: Respir Res. 2020 Aug 7;21:207. doi: 10.1186/s12931-020-01478-x (PMC7412648; doi:10.1186/s12931-020-01478-x)

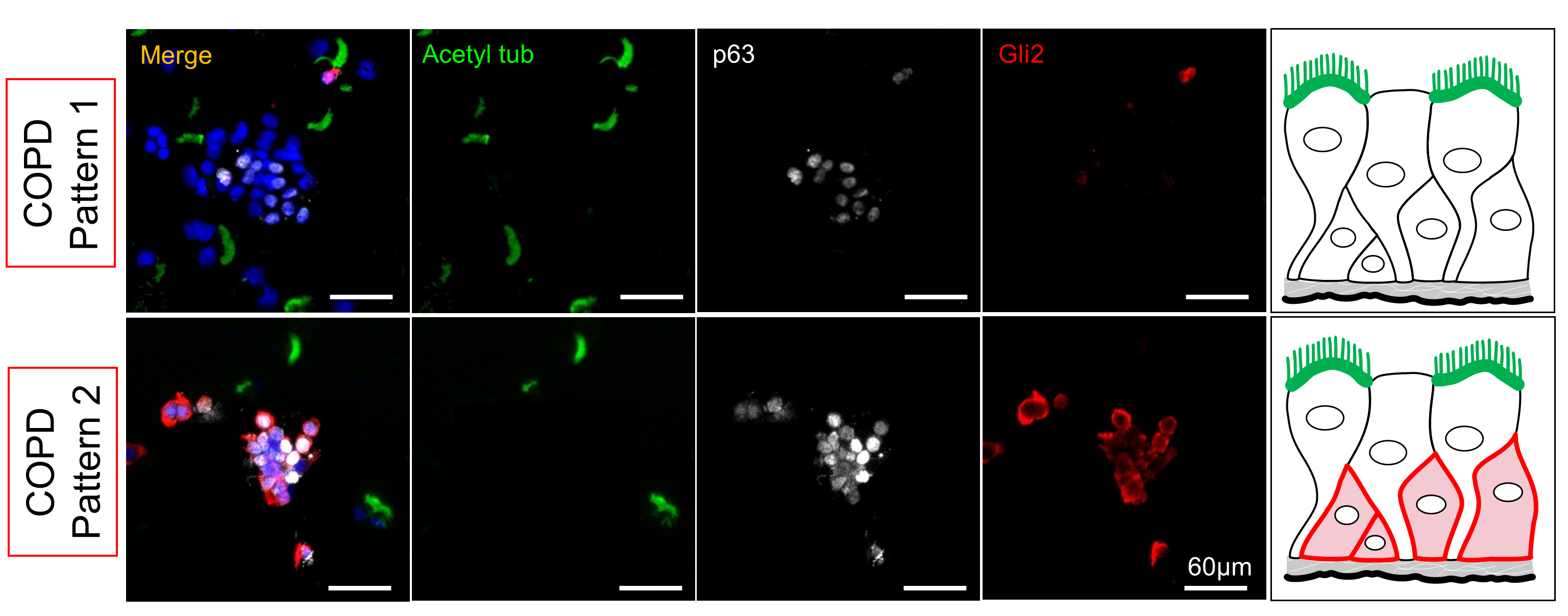

Supplement: Supplementary file 4 — Additional file 4: Figure S1. Heterogeneous Gli2 localization pattern in airway progenitor basal cells from COPD patients. Representative micrograph showing a Region Of Interest of a bronchial brushing stained for cilia (Acetylated tubulin, green); Gli2 (Gli2, red); basal cell (p63, white) and cell nuclei (DAPI, blue) both in COPD. Magnification corresponding to the selected area is shown. Insets depict localization of the Gli2 transcription factor. [file 12931_2020_1478_MOESM4_ESM.tif]

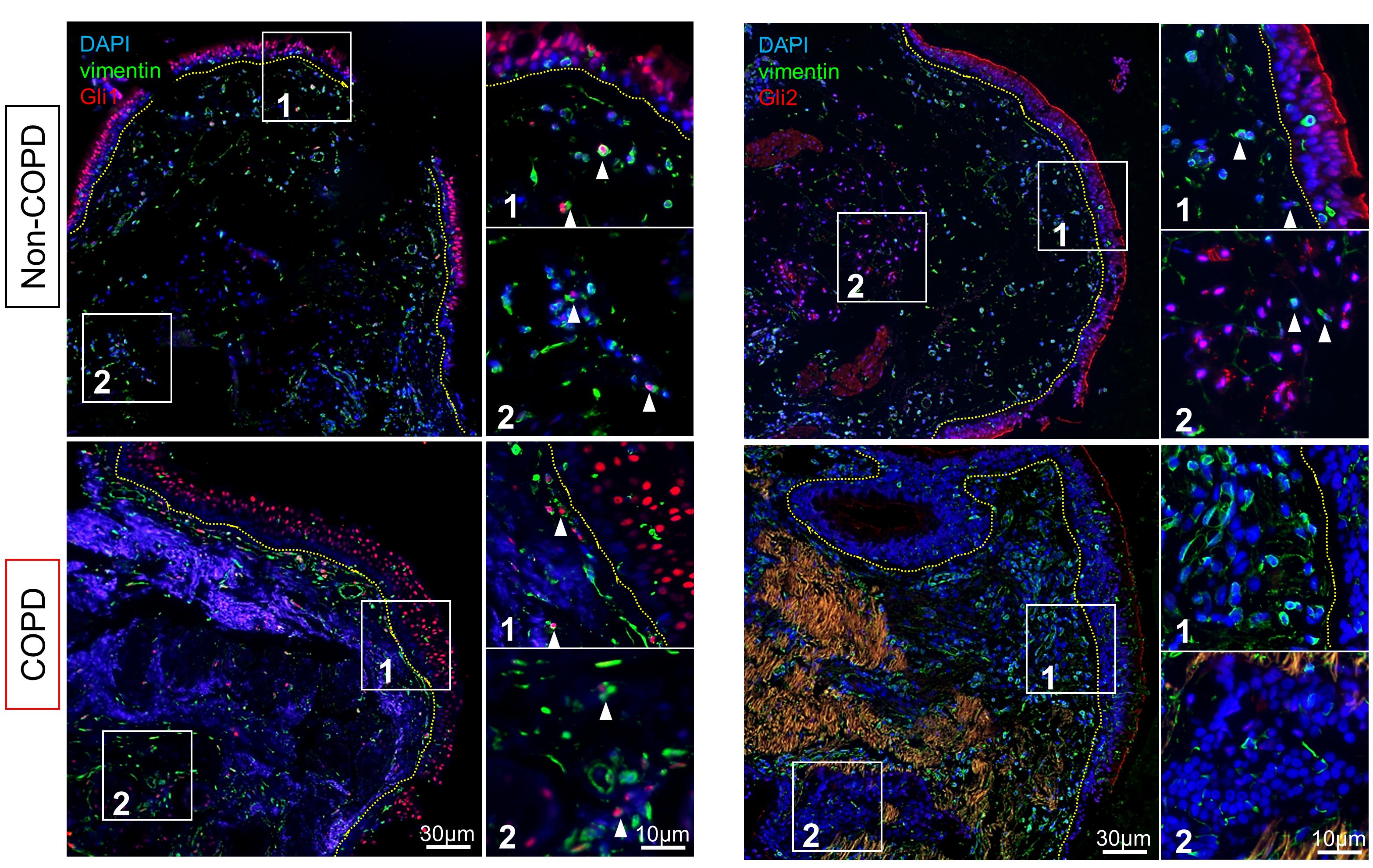

Supplement: Supplementary file 7 — Additional file 7: Figure S4. Peribronchial mesenchymal cells present no alteration of HH signalling in COPD patients. Representative micrograph showing a ROI of a bronchial biopsy stained for mesenchymal cells (vimentin, green); Gli1 (left, red) or Gli2 (right, red); and cell nuclei (DAPI, blue). Magnification corresponding to the selected area is shown. [file 12931_2020_1478_MOESM7_ESM.tif]
